# Development and Characterization of Chromosome Segment Substitution Lines Derived from *Oryza rufipogon* in the Background of the *Oryza sativa* *indica* Restorer Line R974

**DOI:** 10.3390/genes13050735

**Published:** 2022-04-22

**Authors:** Gumu Ding, Biaolin Hu, Yi Zhou, Wanling Yang, Minmin Zhao, Jiankun Xie, Fantao Zhang

**Affiliations:** 1 College of Life Sciences, Jiangxi Normal University, Nanchang 330022, China; dgm2207733038@163.com (G.D.); zhouyi25@mail2.sysu.edu.cn (Y.Z.); zhaomm0222@163.com (M.Z.); 2 Rice National Engineering Laboratory, Rice Research Institute, Jiangxi Academy of Agricultural Sciences, Nanchang 330022, China; hubiaolin992@126.com; 3 Jiangxi Provincial Key Laboratory of Protection and Utilization of Subtropical Plant Resources, Nanchang 330022, China; yangwl3058@163.com

**Keywords:** wild rice, chromosome segment substitution line, germplasm resource, salt stress tolerance, restorer line

## Abstract

Dongxiang wild rice (DXWR) (*O. rufipogon* Griff.), which has the northernmost worldwide distribution of a wild rice species, is a valuable genetic resource with respect to improving stress tolerance in cultivated rice (*Oryza sativa* L.). In the three-line hybrid rice breeding system, restorer lines play important roles in enhancing the tolerance of hybrid rice. However, restorer lines have yet to be used as a genomic background for development of substitution lines carrying DXWR chromosome segments. We developed a set of 84 chromosome segment substitution lines (CSSLs) from a donor parent DXWR × recurrent parent restorer line R974 (*Oryza sativa indica*) cross. On average, each CSSL carried 6.27 introgressed homozygous segments, with 93.37% total genome coverage. Using these CSSLs, we identified a single QTL, *qDYST-1,* associated with salt stress tolerance on chromosome 3. Furthermore, five CSSLs showing strong salt stress tolerance were subjected to whole-genome single-nucleotide polymorphism chip analyses, during which we detected a common substitution segment containing *qDYST-1* in all five CSSLs, thereby implying the validity and efficacy of *qDYST-1*. These novel CSSLs could make a significant contribution to detecting valuable DXWR QTLs, and provide important germplasm resources for breeding novel restorer lines for use in hybrid rice breeding systems.

## 1. Introduction

Rice (*Oryza sativa* L.) is among the most important field crops worldwide, feeding more than half the world’s population [1]. There is growing evidence to indicate that cultivated rice is derived from the domestication of the common wild rice *Oryza rufipogon* Griff. [2], during the process of which, genetic diversity has been progressively reduced, with numerous favorable genes being lost [3]. In contrast, wild rice species represent a natural gene pool for rice improvement, given that they have conserved abundant valuable genetic resources associated with multiple agronomic traits of interest, including grain quality and yield and biotic and abiotic stress tolerance [4]. Consequently, transferring elite genetic resources from wild rice to cultivated rice is viewed as an important strategy for breeding superior rice varieties.

In wild rice and other rice species, the most important agronomic traits are generally controlled by multiple quantitative trait loci (QTLs) [5,6,7], and normally it is not difficult to transfer whole chromosome segments from wild rice to cultivated rice, as these species have the same genome type and are closely related [8]. It is nevertheless notably more difficult to fine map and characterize valuable QTLs associated with the target traits of wild rice species, and overcoming this challenge necessitates the construction and utilization of suitable genetic populations. In previous studies, although numerous temporary mapping populations (such as F_2_ and BC_1_ populations) and permanent primary mapping populations (such as DH and RIL populations) have been widely applied in wild rice species for genetic analysis of complex traits, these populations are generally inadequate for the fine mapping and analysis of individual QTLs, owing to genetic background noise [9,10,11].

However, the use of chromosome segment substitution lines (CSSLs) can effectively eliminate the influence of genetic background noise and thereby enhance the accuracy of QTL mapping and effect analyses. CSSL populations consists of lines carrying the entire genome of the donor parent, although each CSSL carries only a single or, at most, a few different chromosome segments from the donor parent [12]. A significant difference between a single CSSL and the recurrent parent regarding the value of any trait analyzed is assumed to be attributable to the donor-derived substituted chromosome segments. To date, a number of CSSL populations have been constructed, and numerous QTLs have been identified in different rice species. For example, Yuan et al. constructed a CSSL population derived from the rice cultivar 9311 and wild rice DP30, in which they identified numerous QTLs controlling plant architecture, agronomic traits, and cold tolerance [13]. Similarly, Bessho-Uehara et al. developed 40 CSSLs of *Oryza barthii* in the background of the elite *japonica* cultivar Koshihikari and detected multiple QTLs associated with yield-related traits [14], whereas Okada et al. developed a set of 49 CSSLs for the excellent sake-brewing rice Yamadanishiki and a cooking cultivar Koshihikari, among which they detected several QTLs associated with rice grain size [15]. The findings of these studies thus serve to highlight the value of such CSSL populations in genetic analyses with respect to the detection of QTLs associated with complex agronomical traits.

Salinity is currently becoming one of the most significant abiotic stresses affecting crop production and quality [16]. Rice is a salt-sensitive crop, and its growth and development can be greatly affected by salt stress throughout its life. Therefore, it is imperative to identify the QTLs of salt stress tolerance in rice and understand the underlying molecular mechanisms. Singh et al. [17] recently provided a comprehensive list of 935 QTLs from previous studies on salt stress tolerance in rice. Analysis of the most important genomic loci for improving salt stress tolerance by meta-QTL assay found that chromosome 1 had the highest number of related QTLs, followed by chromosomes 3, 2, 6, and 4 [17]. These results provide breeders with abundant genetic resources for breeding salt stress-tolerant rice cultivars.

Among wild rice accessions, *O. rufipogon*, which is highly compatible with *O. sativa*, is widely used for salt stress-tolerant QTL identification and genetic improvement programs to develop salt stress-tolerant rice cultivars [18]. Dongxiang wild rice (DXWR: *O. rufipogon*), which is distributed in Dongxiang County, Jiangxi Province, China, is considered to have the northernmost distribution limit (28°14′ N) of any wild rice species worldwide [19], and previous studies have revealed that this rice has abundant genetic resources associated with tolerance to a range of abiotic stresses, including low and high temperatures, salt, and drought, as well as good seed storability [19,20,21,22]. To date, geneticists and breeders have constructed multiple types of mapping populations to examine and utilize the valuable genetic resources of DXWR, among which the development of CSSL populations has received considerable attention. Qiao et al., for example, constructed a set of 198 CSSLs derived from a cross between the donor parent DXWR and the recurrent parent 9311 (*Oryza sativa* L. subsp. *indica*), with substitute segments in the CSSLs covering 84.90% of the DXWR genome [8]. More recently, Ma et al. have developed 104 DXWR CSSLs in the background of the Nipponbare rice cultivar (*O. sativa* L. subsp. *japonica*), with the CSSLs covering 87.94% of the DXWR genome [23].

In the three-line hybrid rice breeding system, restorer lines play important roles in contributing to the improvement of hybrid rice agronomic traits [24], and consequently, breeding restorer lines with high combining ability, high quality, and stress tolerance has emerged as one of the vital research directions. To promote the application of the elite genetic resources of DXWR in restorer line improvement, it is of particular importance to construct DXWR–restorer line cultivar rice CSSLs. However, to the best of our knowledge, there have to-date been no CSSLs’ development for DXWR that use a restorer line cultivar rice background. In this study, we accordingly used DXWR as the donor and an elite restorer line, R974, as the recurrent parent to construct a set of CSSLs. The CSSLs were genotyped based on an analysis of 140 polymorphic markers, and collectively, the constructed CSSLs, which had an average substituted segment length of 16.48 cM (centimorgan), covered 93.37% of the DXWR genome. By subjecting these CSSLs to salt stress, we identified a single QTL, *qDYST-1*, associated with salt stress tolerance. Moreover, the findings of a whole-genome single-nucleotide polymorphism (SNP) chip analysis confirmed the validity and efficacy of *qDYST-1* with respect to five CSSLs showing strong salt tolerance. These CSSLs could make a valuable contribution to the identification of elite genetic resources from DXWR and provide a solid basis for enhancing restorer lines for utilization in hybrid rice breeding systems.

## 2. Materials and Methods

### 2.1. Plant Materials

For the purposes of the present study, we used DXWR, a common wild rice found growing in Dongxiang County, Jiangxi Province, China, as the donor parent and R974, a representative restorer line, as the recurrent parent. The accession of DXWR, used in this study, was obtained from the in situ population of Zhangtang [25]. The seeds of DXWR and R974 were provided by the Rice Research Institute, Jiangxi Academy of Agricultural Sciences, China.

### 2.2. DNA Extraction and PCR

Total genomic DNA was extracted from the fresh leaves of the plants using the CTAB method [26], and used as a template for subsequent PCR amplification, performed using 10 μL reaction mixtures containing 1.0 μL of DNA, 0.5 μL of each forward and reverse primer, 5.0 μL of 2 × Fast Taq Premix, and 3.0 μL of ddH_2_O. The PCR program consisted of an initial denaturation step at 95 °C for 5 min, followed by 32 cycles at 95 °C for 30 s, 56 °C for 30 s, and 72 °C for 30 s, and a final extension of 72 °C for 5 min. The PCR products were visualized by electrophoresis on 8% polyacrylamide gels, followed by silver staining [27].

### 2.3. Construction of CSSLs

CSSLs were constructed by initially crossing DXWR with the restorer line R974 to produce an F_1_ hybrid generation. F_1_ individuals were backcrossed to the recurrent parent R974 to produce BC_1_F_1_ plants, and by conducting subsequent backcrossing and successive self-crossings, we obtained 220 BC_3_F_7_ lines. A linkage map for these was constructed using Mapmaker/Exp software [28]. The distance between molecular markers was evaluated using the Kosambi function and presented in centiMorgans (cM) based on the genotyping of lines. Furthermore, for each line, we analyzed the length and proportion of the substitute segments derived from the donor parent. Those lines containing more than 90% of the R974 genome were subsequently selected to construct the CSSL population using CSSL finder software (http://mapdisto.free.fr/CSSLFinder/) (accessed on 1 November 2021) [29], and graphical genotyping analysis of the selected lines was performed using GGT software (https://ggt.software.informer.com/, accessed on 1 November 2021) [30].

### 2.4. Evaluation of Salt Stress Tolerance

Evaluation of the salt stress tolerance of CSSL plants was performed at the seedling stage. The seeds of each CSSL were surface-sterilized, germinated in a moistened Petri dish, and thereafter grown in a plant hydroponic box exposed to a 14 h:10 h (26 °C/24 °C) light:dark photoperiod. The seedlings were cultured in Kimura B nutrient salt solution (Coolaber Technology Co., LTD., Beijing, China), and on reaching the four-leaf stage were treated with 200 mM NaCl for 7 days. Having undergone the salt stress treatment, the seedlings were then recovered by replacing the salt solution with the normal nutrient solution for 7 days and renewing the solution at 3 day intervals, after which the survival rate was assessed and used for phenotyping the salt stress tolerance of plants. The experiment was performed as two independent replicates.

### 2.5. QTL Analysis and SNP Chip Assay

QTL analysis based on inclusive composite interval mapping was performed using the additive QTL (ICIM-ADD) mapping method of QTL IciMapping software (version 4.2), which enables the mapping of CSSL QTLs [31]. The threshold logarithm of odds (LOD) score was set to 2.0, and other parameters were set to the default values recommended in the software user manual. Genotyping of five selected salt stress-tolerant CSSLs and the two parents was performed by Wuhan Greenfafa Institute of Novel Genechip R&D Co., Ltd., (Greenfafa, Wuhan, China) using an Illumina GSR40K SNP chip containing 44263 SNPs (https://www.greenfafa.com, accessed on 1 November 2021). SNP loci were determined based on the resequencing of 4726 worldwide rice cultivars, and the chromosomal locations of these SNPs were determined from the Nipponbare MSU7.0 reference genome.

## 3. Results

### 3.1. Screening of Polymorphic Markers and Linkage Map Construction

To determine polymorphisms between the DXWR and R974 parents, we used a total of 625 molecular markers distributed across the 12 rice chromosomes, among which, 140 showed a clear polymorphism. These polymorphic markers were found to be unevenly distributed on the 12 chromosomes, ranging from 8 on chromosome 11 to 20 on chromosome 1, with an average of 11.67 markers per chromosome (Table 1). Details of the polymorphic markers are listed in Supplementary Table S1. These markers were subsequently used to analyze the genotypes of the CSSLs and for the construction of a linkage map, which spanned 1556.30 cM of the 12 chromosomes, with an average distance of 11.12 cM between two adjacent markers, ranging from 6.48 cM (chromosome 7) to 14.24 cM (chromosome 5) (Table 1).

### 3.2. Development and Characterization of the CSSL Population

**Figure 1:**
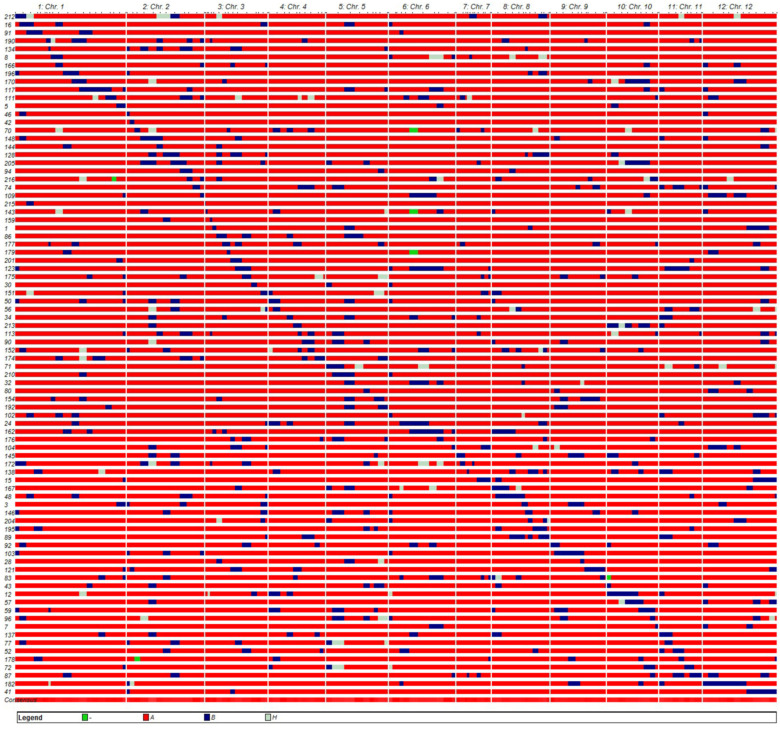
A graphical representation of the genotypes of the chromosome segment substitution line (CSSL) population. Regions with red, blue, and gray backgrounds indicate homozygous segments from the recurrent parent R974, the donor parent DXWR, and the two parents, respectively. The green regions indicate missing segments. Each row represents a single CSSL.

**Figure 2:**
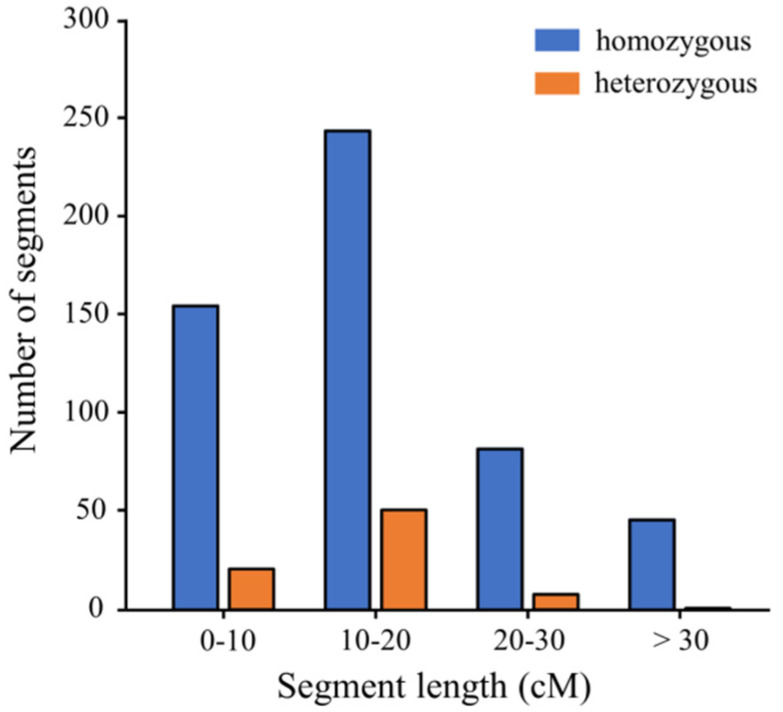
Distribution of the lengths of substituted chromosome segments in the chromosome segment substitution line population.

To ensure maximal possible coverage of the DXWR genome, we removed those CSSLs with repetitive insertions or an excessive number of segments, and having done this, we selected 149 CSSLs containing more than 90% of the R974 genome, according to the genotypes. Among these 149 CSSLs, 84 with appropriate substitution segments were selected using CSSL finder software and arranged in order according to the position of the substitution chromosome segments. Thereafter, to develop a CSSL population, we subjected the selected CSSLs to graphical genotyping analysis using GGT software (Figure 1). In total, the CSSL population thus obtained carried 527 homozygous and 81 heterozygous chromosome segments from the donor parent DXWR (Table 2). Given the relatively low number of heterozygous segments and the fact that most of these overlapped with the substitution regions of the homozygous segments, we decided to focus on analyzing the homozygous substitution segments. The length of the homozygous substituted segments in this CSSL population ranged from 1.70 (on Chr. 3 of CSSL215) to 90.30 cM (on Chr. 12 of CSSL182). Furthermore, 28.95% of the substitution segments were found to be smaller than 10 cM, whereas 48.52% were between 10 and 20 cM in size, 14.80% were between 20 and 30 cM, and 7.73% exceeded 30 cM (Figure 2). Among these CSSLs, CSSL42 carried only a single homozygous substitution segment of length 10 cM located on chromosome 2. On average, each CSSL carried 6.27 homozygous substituted segments of average length 16.48 cM, and collectively, these segments covered 93.37% (1453.05 cM) of the entire DXWR genome.

We also established that the substitution segments were unevenly distributed, with the highest (82) and lowest (25) number of segments mapping to chromosomes 1 and 7, respectively. Furthermore, introduction of the substitution chromosome segments was found to differ from chromosome to chromosome, with homozygous substitution segment coverages ranging from only 78.02% on chromosome 6 up to 100% on chromosomes 2, 8, 9, 10, 11, and 12 (Table 2).

### 3.3. QTL Mapping for Salt Stress Tolerance

**Figure 3:**
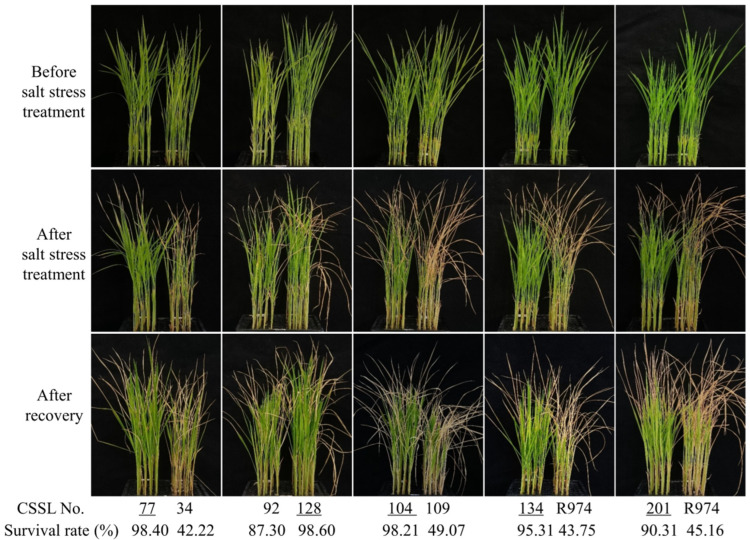
Phenotypic comparison between selected chromosome segment substitution lines (CSSLs) and the recurrent parent R974 prior to salt stress exposure, after salt stress treatment, and after recovery. The CSSLs with underline were selected to single-nucleotide polymorphism chip assay.

**Figure 4:**
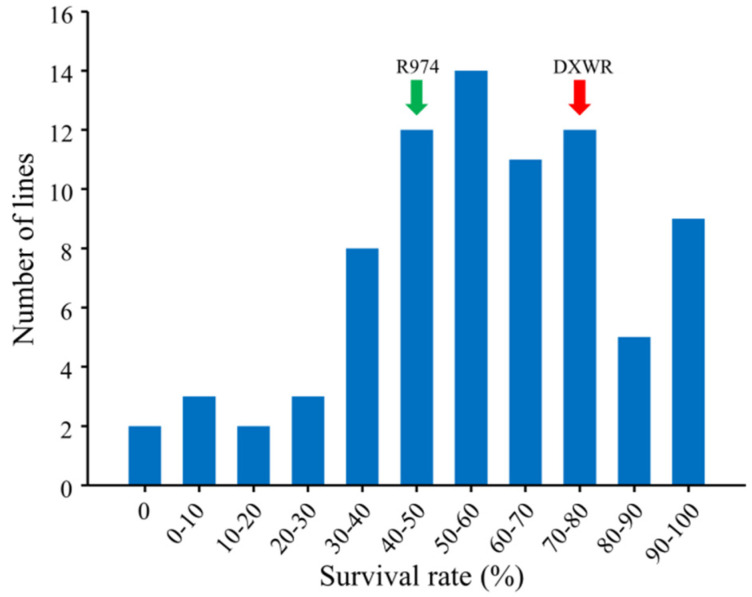
Survival rates of chromosome segment substitution line seedlings subjected to 200 mM NaCl stress. The data represents the average of two independent experiments.

The salt stress tolerance of the CSSL population and parental plants was evaluated at the seedling stage. Prior to subjecting the seedlings to salt stress treatment, we detected no significant differences with respect to the performance of the CSSLs. However, having exposed the CSSLs to salt stress for 7 days, we observed a wide range of phenotypic variation among these lines. Similarly, having allowed the treated plants to recover in normal nutrient solution for 7 days, we noted a considerable variation in CSSL survival, ranging from 0 to 98.60%, whereas comparatively, 43.75% of the plants of the recurrent parent R974 survived (Figure 3 and Figure 4). As a consequence of these analyses, in both independent experiments, we detected a single QTL, *qDYST-1*, located between markers chr3-58.7 and chr3-68.6 on chromosome 3, which was found to be associated with an enhancement of salt stress tolerance. In the two independent experiments, the phenotypic variation explained by *qDYST-1* was 11.23 and 12.56%. In addition, *qDYST-1* had an additive effect of 0.16 and 0.15 for increasing survival rates, derived from the DXWR allele.

### 3.4. SNP Chip Assay

**Figure 5:**
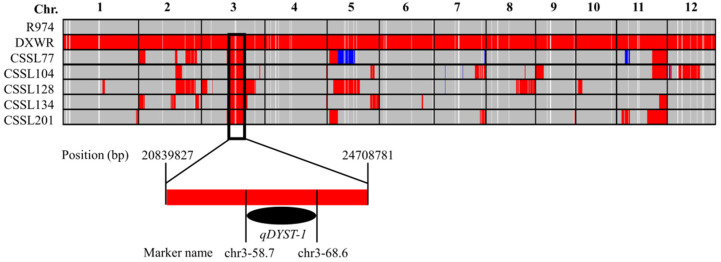
Genotyping of the two parents and five salt stress-tolerant chromosome segment substitution lines (CSSLs) based on SNP chip analysis. The black frame represents the common chromosome segment identified in the five CSSLs. This segment completely encompassed the *qDYST-1* locus.

To confirm the results of QTL mapping, five CSSLs (CSSL77, 104, 128, 134, and 201) with strong salt stress tolerance were selected for further SNP detection via whole-genome SNP chip screening. SNP genotyping was performed using an Illumina GSR40K SNP chip containing 44,263 SNPs. We accordingly screened out a total of 32,887 high-quality SNP loci for further analysis, of which 32,595 had positional coordinates. Given that the heterozygous genotypes of parents will segregate among their offspring and that it is not possible to trace their origins, we thus used differential homozygous loci between the two parents for genotyping the five CSSLs. As shown in Figure 5, in all of the five CSSLs, we identified a single common chromosome segment extending from base pair 20,839,827 to 24,708,781 bp on chromosome 3, which completely encompassed the *qDYST-1* locus (Figure 5). On the basis of these findings, we tentatively assumed that *qDYST-1* could be the causal locus associated with the observed salt stress tolerance of DXWR.

## 4. Discussion

Over the past several decades, numerous CSSL populations have been constructed in rice and other crops [32,33,34,35]. In an ideal CSSL population, each line contains only a single or a few substitution chromosome segments derived from the donor parent in a uniform genetic background of the recurrent parent [12]. The simple genomic structure of CSSLs is advantageous in that it facilitates the identification of causal loci associated with complex agronomic traits and enables us to analyze correlations between traits and substitution segments. To date, CSSLs have made significant contributions not only in genetic studies but also crop breeding programs [35,36].

In the 1970s, Professor Yuan Longping and his assistants discovered an accession of wild rice with male sterility, which they used to breed three-line hybrid rice varieties [37,38]. To date, three-line hybrid rice has made a considerable contribution to enhancing rice production both in China and worldwide [39]. Three-line hybrid rice systems consists of a sterile line, a maintainer line, and a restorer line, among which the restorer line is a vital factor with respect to improving the agronomic traits of hybrid rice. However, few CSSLs have been reported for wild rice, in which a rice cultivar is used as a restorer line background.

In China, R974 is a representative restorer line utilized in three-line hybrid rice breeding [40]. DXWR is characterized by strong tolerance to multiple biotic and abiotic stresses and accordingly constitutes a valuable natural gene pool for the improvement of cultivated rice [19,20,21,22,23]. In the present study, we developed a CSSL population derived from DXWR in the genetic background of the R974 restorer line and found that each CSSL carried an average of 6.27 homozygous substitution segments. Using the same wild rice accession DXWR as the donor parent, Qiao et al. (2016) developed CSSLs in the genetic background of the 9311 cultivar of *O. sativa indica* and found that each CSSL carried 2.16 substitution segments [8]. Similarly, using a *japonica* rice cultivar Nipponbare background, Ma et al. (2019) constructed CSSLs, each of which containing an average of four substitution segments [23]. Comparatively, in the present study, we obtained a larger number of substitution segments, which can be attributed to the fact that the generation of backcrossing is not enough. Consequently, we believe that in the future, more efforts should be devoted to advanced backcrossing and marker-assisted selection.

Salinity is among the most devastating abiotic stresses constraining rice production [41], and against a current background of increasing salinity levels worldwide, gaining a more complete understanding of the mechanisms underlying salt stress tolerance and breeding salt stress-tolerant rice cultivars is becoming ever more important. However, although some progress has been made to date in this regard, the mechanisms associated with salt stress tolerance in rice continue to remain poorly elucidated [42]. In this context, identifying a wider array of genetic sources associated with salt stress tolerance from different rice species is of particular value. Given that DXWR is characterized by strong salt stress tolerance, it is considered to be an ideal material that can be systematically mined to identify salt stress tolerance QTLs and accordingly examined to determine the underlying mechanisms [43]. However, to date, limited QTLs have been detected in DXWR. Quan et al. [18] found nine QTLs for salt stress tolerance in DXWR at the seedling stage, located on chromosomes 1, 3, 4, 5, 6, 8, and 10. Among these, one QTL, *qST3*, was located on chromosome 3 with a physical location of 6.2 Mb [18]. In the present study, we succeeded in mapping a putative salt stress tolerance-associated QTL, designated *qDYST-1,* to an interval between the two markers chr3-58.7 and chr3-68.6 (with a physical distance of 22.17 Mb to 23.89 Mb) on chromosome 3. The location of *qDYST-1* did not overlap with *qST3*, indicating that *qDYST-1* is a novel QTL involved in the salt stress tolerance of DXWR.

Whole-genome sequencing has been successfully applied in rice breeding studies, such as genetic resource development, QTL mapping, genotype homozygosity analysis, and background genome selection [44,45]. In this study, the results of whole-genome SNP chip analyses revealed that five selected CSSLs with strong salt stress tolerance had a single common chromosome segment that completely encompassed the *qDYST-1* locus, thereby indicating that *qDYST-1* could be the causal locus associated with the salt stress tolerance trait of DXWR. We thus believe that these five CSSLs constitute important germplasm resources that stand to make a valuable contribution to the detection of QTLs of interest in DXWR and the breeding of salt stress-tolerant hybrid rice. In this regard, Zhou et al. (2016) analyzed the transcriptome profiles of DXWR and detected a large number of transcripts that were differentially expressed in the response to salt stress [21]. Similarly, on the basis of the comparative analysis performed in the present study, we identified 22 and 15 transcripts in the interval containing *qDYST-1* that were differentially expressed in the leaves and roots of rice, respectively (Table S2). Among these differentially expressed transcripts, one (*LOC_Os03g40070.1*) encoded a transposon protein and another (*LOC_Os03g39940.1*) encoded a retrotransposon protein. Previous studies have shown that transposon/retrotransposon proteins confer salt stress tolerance in various plants [46,47]. Our data, combined with previous studies, suggest that plants could have a common molecular mechanism regulated by transposon/retrotransposon proteins to respond to salt stress. Numerous studies have reported that zinc finger proteins play vital roles in plant tolerance to salt stress [48,49]. Two differentially expressed transcripts (*LOC_Os03g42200.1* and *LOC_Os03g41110.1*), both encoding zinc finger proteins, were found in the interval region containing *qDYST-1*, implying that they could be involved in the rapid response of DXWR to salt stress stimuli. In addition, we also found three transcripts (*LOC_Os03g42240.1*, *LOC_Os03g42280.1*, and *LOC_Os03g42420.1*) that encoded B3 DNA-binding domain-containing proteins. This result is consistent with previous observations that B3 DNA-binding domain-containing proteins play important roles in the adaptation to salt stress [16,50]. These findings will provide a valuable basis for further fine-scale mapping and cloning of salt stress tolerance-related genes in DXWR.

## 5. Conclusions

In this study, we constructed the first set of chromosome segment substitution lines derived from wild rice in the genetic background of a cultivar restorer line. In this population, the homozygous segments collectively covered 93.37% of the entire DXWR genome, with each CSSL harboring an average of 6.27 homozygous substitution chromosome segments. Using this CSSL population, we detected a single QTL, *qDYST-1,* that appears to be associated with tolerance to salt stress. Moreover, genotyping based on SNP chip analysis confirmed the validity and efficacy of the *qDYST-1* locus. Consequently, we believe that this CSSL population could be used to identify further QTLs that control other traits of interest in DXWR, and could be used directly to breed new restorer lines for hybrid rice improvement.

## Figures and Tables

**Table 1:** Distribution of polymorphic markers on the 12 rice chromosomes.

Chr.	Chr. Length (cM)	No. of Markers	Average Distance between Adjacent Markers (cM)
1	229.70	20	11.49
2	160.30	12	13.36
3	128.70	15	8.58
4	117.70	11	10.70
5	128.20	9	14.24
6	137.20	11	12.47
7	71.30	11	6.48
8	119.50	12	9.96
9	115.30	11	10.48
10	105.90	9	11.77
11	87.80	8	10.98
12	154.70	11	14.06
Total	1556.30	140	11.12

**Table 2:** Substitution segments of Dongxiang wild rice in a chromosome segment substitution line population, and the cumulative proportion of donor genome represented by homozygous and heterozygous segments.

Chr.	Homozygous Segments			Heterozygous Segments			Total Segment Length (cM)	Effective Coverage Length (cM) (Homo)	Genome Coverage (%) (Homo)
	Number of Segments	Total Segment Length (cM)	Average Length (cM)	Number of Segments	Total Segment Length (cM)	Average Length (cM)			
1	70	1104.40	15.78	12	165.70	13.81	1270.10	222.60	96.91
2	64	1018.60	15.92	8	139.05	17.38	1157.65	160.30	100.00
3	55	688.75	12.52	5	53.00	10.60	741.75	117.15	91.03
4	36	569.30	15.81	4	49.95	12.49	619.25	105.00	89.21
5	47	885.05	18.83	10	170.70	17.07	1055.75	101.70	79.33
6	42	769.55	18.32	9	143.35	15.93	912.90	107.05	78.02
7	24	233.80	9.74	1	10.05	10.05	243.85	56.05	78.61
8	45	715.60	15.90	10	117.85	11.79	833.45	119.50	100.00
9	28	554.45	19.80	3	23.75	7.92	578.20	115.30	100.00
10	35	631.90	18.05	8	113.50	14.19	745.40	105.90	100.00
11	32	518.40	16.20	2	29.35	14.68	547.75	87.80	100.00
12	49	993.70	20.28	9	92.65	10.29	1086.35	154.70	100.00
Average	43.92	723.63		6.75	92.41				92.76
Total	527	8683.50	16.48	81	1108.90	13.69	9792.40	1453.05	93.37

## Data Availability

Not applicable.

## Supplementary Materials

The following supporting information can be downloaded at: https://www.mdpi.com/article/10.3390/genes13050735/s1, Table S1. The details of the polymorphic markers used in this study. Table S2. The candidate transcripts within the interval containing *qDYST-1*, identified by differential expression combined analysis.
